# Nephroureterectomy increase 5 year survival in patients on dialysis with upper urinary tract urothelial carcinoma

**DOI:** 10.18632/oncotarget.20180

**Published:** 2017-08-11

**Authors:** Cih-En Huang, Yao-Hsu Yang, Wen-Cheng Chen, Kuo-Tsai Huang, Pau-Chung Chen, Ying-Huang Tsai, Wei-Yu Lin

**Affiliations:** ^1^ Division of Hematology Oncology, Department of Medicine, Chang Gung Memorial Hospital, Chiayi, Taiwan; ^2^ Graduate Institute of Clinical Medical Sciences, Chang Gung University, Taoyuan, Taiwan; ^3^ Chang Gung University of Science and Technology, Chiayi, Taiwan; ^4^ Traditional Chinese Medicine, Chang Gung Memorial Hospital, Chiayi, Taiwan; ^5^ Center of Excellence for Chang Gung Research Datalink, Chang Gung Memorial Hospital, Chiayi, Taiwan; ^6^ School of Traditional Chinese Medicine, Chang Gung University, Taoyuan, Taiwan; ^7^ Department of Radiation Oncology, Chang Gung Memorial Hospital, Chiayi, Taiwan; ^8^ College of Medicine, Chang Gung University, Taoyuan, Taiwan; ^9^ Division of Urology, Department of Surgery, Chang Gung Memorial Hospital, Chiayi, Taiwan; ^10^ Institute of Occupational Medicine and Industrial Hygiene, National Taiwan University College of Public Health, Taipei, Taiwan; ^11^ Department of Public Health, National Taiwan University College of Public Health, Taipei, Taiwan; ^12^ Department of Environmental and Occupational Medicine, National Taiwan University Hospital and National Taiwan University College of Medicine, Taipei, Taiwan; ^13^ Division of Pulmonary and Critical Care Medicine, Department of Respiratory Care, Chang Gung Memorial Hospital, Chiayi, Taiwan; ^14^ Department of Respiratory Therapy, Chang Gung University, Taoyuan, Taiwan

**Keywords:** nephroureterectomy, dialysis, elderly, upper tract urothelium carcinoma

## Abstract

**Background:**

There is a high incidence rate of upper tract urothelial carcinoma (UTUC) in patients on dialysis. However, the studies about nephroureterectomy (NU) in this high surgical risk group are limited. The aim of this study is to investigate the outcomes of NU in this population.

**Results:**

There were total 931 patients enrolled and 218, 582, 131 patients were non-NU, unilateral and one-stage bilateral NU, respectively. NU provided better 5-year overall survival (66% versus 51% in non-NU, *P* = 0.001). 19.7% of patients with unilateral NU had successive contralateral NU with a mean interval period of 695 days. Even for the elderly, there were no significant difference in duration of hospitalization, 30- and 90-day mortality between unilateral and bilateral NU.

**Materials and Methods:**

Patients on dialysis with UTUC between January 1998 and December 2012 were assessed from the nationwide cohort of Taiwan National Health Insurance Research Database. We classified these patients into non-NU and NU groups. In NU group, we analyzed clinical outcomes of patient groups between different NU types and surgical methods.

**Conclusions:**

Although the high surgical risk in patients on dialysis with UTUC, NU provided better 5-year overall survival. One-stage bilateral NU both provides comparable safety profile and avoids 19.7% of successive contralateral NU in less than two years. Even in the elderly, one-stage bilateral NU is safe and feasible.

## INTRODUCTION

Compared to other countries, Taiwan has a remarkably high incidence and prevalence of patients with end-stage renal disease (ESRD) [1]. Increased cancer risk in such patients under chronic dialysis has been well documented in various studies and urologic cancer is the most common. In Western countries and Japan, the most prevalent urologic malignancy in patients on hemodialysis is renal cell carcinoma (RCC) [2–5]. In contrast, the most prevalent in patients with ESRD on dialysis or receiving kidney transplantation in Taiwan is urothelial carcinoma (UC), with incidence among 0.89 to 1.69% [6, 7].

The pathogenesis of UC in ESRD patients is unclear. Some studies suggest the impact of analgesics, arsenic-containing groundwater [8], and certain herbs such as aristolochic acid [9]. Aristolochic acid is especially reported to induce upper urinary tract urothelial carcinoma (UTUC) [10]. In Taiwan, the ratio of lower urinary tract to upper urinary tract urothelial carcinoma in ESRD patients is around 6:5, with relatively higher proportion of UTUC compared to other area [11, 12]. The pathological characteristics and clinical presentation of UTUC in patients on dialysis are different from those with normal kidney function, suggesting that UTUC in ESRD patients is a distinct entity in UC [13, 14].

However, data on the prognosis and clinical outcomes of UTUC after hemodialysis is rare, with only case series analyses from single institutes [13, 14]. There is paucity of data regarding surgical outcomes and recommendations for management. This study therefore used the large database from the National Health Insurance to analyze the clinical outcomes, morbidity and mortality of different nephroureterectomy (NU) surgical methods among patients on hemodialysis with UTUC.

## RESULTS

In the study period, there were 931 patients on dialysis and with newly diagnosed UTUC. Among them, 218 did not undergo NU, 582 underwent unilateral NU, and 131 had bilateral NU (Table 1). The median following time of unilateral and bilateral NU were 3.9 and 3.8 years respectively. In the 582 patients who underwent unilateral NU, 115 (19.76%) had contralateral UTUC and subsequently underwent a second NU. The mean interval period between the two successive NU procedures was 695 days.

**Table 1:** Demographic and clinical characteristics of patient on dialysis with UTUC

Treatment	No of patients, *n* = 931	Non-NU, *n* = 218	NU, *n* = 713		*p* value*
			Unilateral (*n* = 582)	Bilateral (*n* = 131)	
Sex					0.387
M	644 (69.2%)	155 (71.1%)	395 (67.9%)	94 (71.8%)	
F	287 (30.8%)	63 (28.9%)	187 (32.1%)	37 (28.2%)	
Age at ESRD					0.001
< 65	663 (71.2%)	134 (61.5%)	417 (71.6%)	112 (85.5%)	
≥ 65	268 (28.8%)	84 (38.5%)	165 (28.4%)	19 (14.5%)	
Median	56.6	59.9	56.5	51.6	< 0.001
Age at cancer diagnosis					0.003
< 65	575 (61.8%)	116 (53.2%)	360 (61.9%)	99 (75.6%)	
≥ 65	356 (38.2%)	102 (46.8%)	222 (38.1%)	32 (24.4%)	
Median	61.4	63.9	61.3	59.2	0.020
Duration of ESRD before cancer					< 0.001
< 1 year	213 (22.9%)	56 (25.7%)	143 (24.6%)	14 (10.7%)	
≥ 1 and < 5 years	338 (36.3%)	99 (45.4%)	205 (35.2%)	34 (26.0%)	
≥ 5 and < 10 years	260 (27.9%)	46 (21.1%)	157 (27.0%)	57 (43.5%)	
≥ 10 years	120 (12.9%)	17 (7.8%)	77 (13.2%)	26 (19.8%)	
Median	3.7	2.3	3.5	6.2	< 0.001
Ever used PD	108 (11.6%)	20 (9.2%)	70 (12.0%)	18 (13.7%)	0.590
Ever RT	111 (11.9%)	8 (3.7%)	69 (11.9%)	34 (26.0%)	< 0.001
CCI(S.D.)	3.8 (2.1)	3.9 (2.2)	3.8 (2.0)	4.0 (2.0)	0.182
Co-morbidity					
Diabetes	267 (28.7%)	63 (28.9%)	168 (28.9%)	36 (27.5%)	0.751
Hypertension	723 (77.7%)	155 (71.1%)	459 (78.9%)	109 (83.2%)	0.265
Tuberculosis	40 (4.3%)	9 (4.1%)	22 (3.8%)	9 (6.9%)	0.117
Hyperlipidemia	267 (28.7%)	56 (25.7%)	167 (28.7%)	44 (33.6%)	0.268
Chronic hepatitis B	68 (7.3%)	8 (3.7%)	43 (7.4%)	17 (13.0%)	0.037
Chronic hepatitis C	78 (8.4%)	9 (4.1%)	50 (8.6%)	19 (14.5%)	0.039
Glomerulonephritis	566 (60.8%)	116 (53.2%)	369 (63.4%)	81 (61.8%)	0.737

Abbreviations: UTUC, upper urinary tract urothelial carcinoma; NU, nephroureterectomy; ESRD, end-stage renal disease; PD, peritoneal dialysis; CCI, Charlson co-morbidity index; RT, renal transplantation.

*p value estimate of Chi-square test or *t* test between unilateral and bilateral NU.

Median age at diagnosis of ESRD and UTUC in the patients with bilateral NU were younger than the unilateral NU group. Duration of ESRD before diagnosis of UTUC was much longer in the bilateral NU group (median duration 6.2 years versus 3.5 years in unilateral NU group. *p* < 0.001) (Table 1). In addition, there was significantly higher proportion of patients receiving renal transplantation in the bilateral NU group than that in unilateral NU group (26% versus 11.9%, respectively, *p* < 0.001). Otherwise, there were no significant differences in gender and history of peritoneal hemodialysis. But male gender was predominant in both groups. There were no differences in CCI and co-morbidities including diabetes, hypertension, tuberculosis, hyperlipidemia, and glomerulonephritis between the two groups except chronic hepatitis B and C. The patients receiving bilateral NU had significantly higher percentage of chronic hepatitis B and C compared to the unilateral NU group (13% versus 7.4% and 14.5% versus 8.6%, respectively).

There were no differences in duration of hospitalization and in 30- and 90-day mortality among patients with unilateral versus bilateral NU and those with open versus laparoscopic NU (Table 2). In the subgroup analysis of patients older than 65 years also showed the similar results. In the patients younger than 65 years old, 30-day mortality were zero in both unilateral and bilateral NU groups, though patients with bilateral NU had higher 90-day mortality rate.

**Table 2:** Surgical outcomes of patients by different treatment and different age groups

	Total number	Hospitalization Days (SD)	Mortality			
			30 days		90 days	
			Dead	*p*-value*	Dead	*p*-value*
**All ages**						
Nephroureterectomy				0.360		0.408
Unilateral	582	14.6 (10.4)	7		17	
Bilateral	131	15.7 (10.8)	0		6	
Operation Methods				1.0000		0.067
Open	491	15.7 (10.9)	5		20	
Laparoscopic	222	13.0 (9.1)	2		3	
**Age < 65 years**						
Nephroureterectomy						0.002
Unilateral	357	13.7 (9.0)	0		1	
Bilateral	99	15.2 (10.5)	0		5	
Operation methods						0.192
Open	328	14.8 (10)	0		6	
Laparoscopic	128	11.9 (6.8)	0		0	
**Age ≥ 65 years**						
Nephroureterectomy				0.602		0.704
Unilateral	225	16.2 (12.2)	7		16	
Bilateral	32	17.3 (11.8)	0		1	
Operation methods				1.000		0.120
Open	163	17.5 (12.4)	5		14	
Laparoscopic	94	14.4 (11.4)	2		3	

*p value estimate of Fisher exact test.

age: at nephron-ureterectomy.

**Figure 1:**
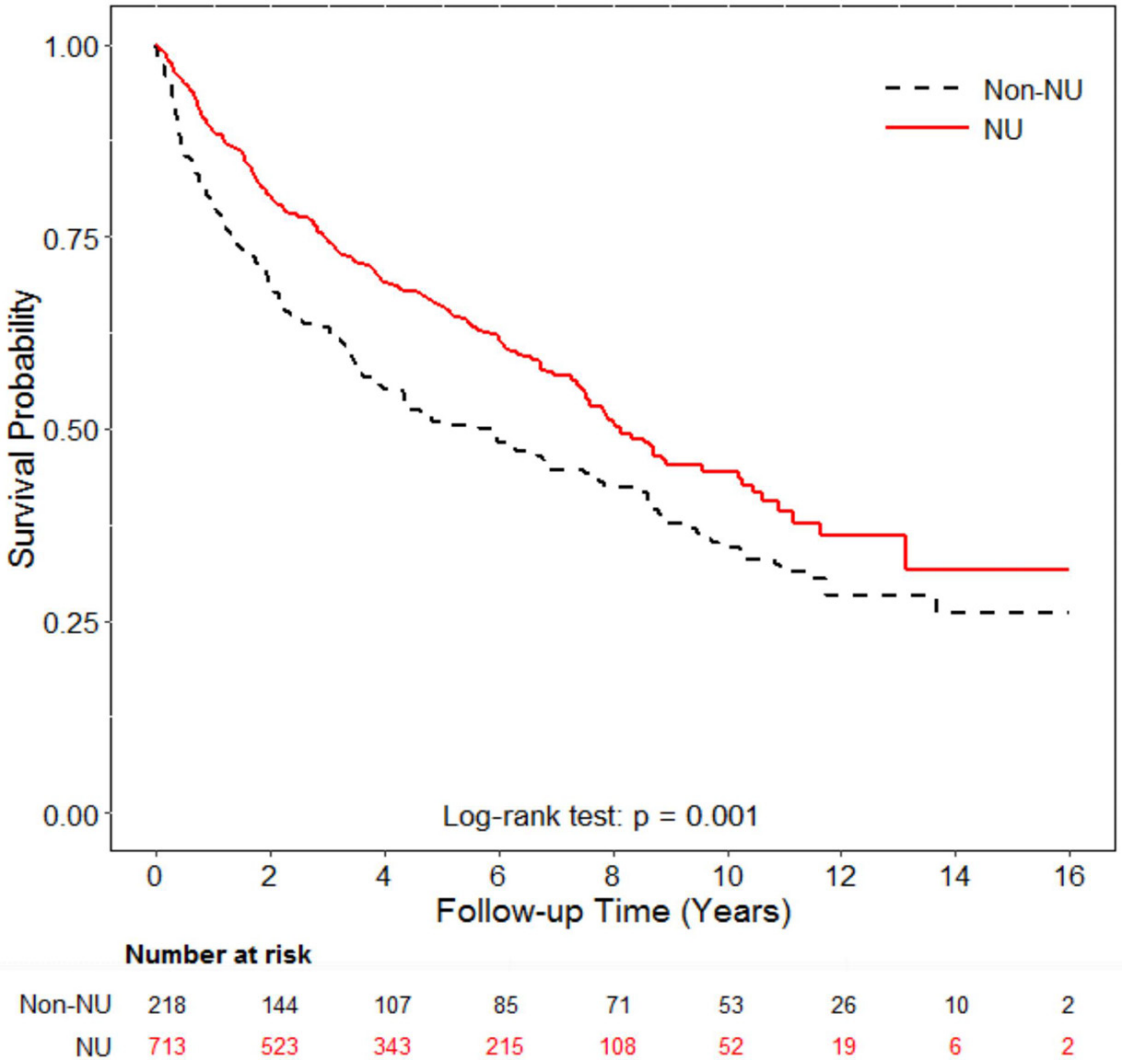
Overall survival analysis of patients with or without nephroureterectomy Patients who underwent nephro-uretectomy (NU) had significantly better five-year overall survival compared to patients without NU (66% vs. 51%; *p* = 0.001, by log-rank test).

**Figure 2:**
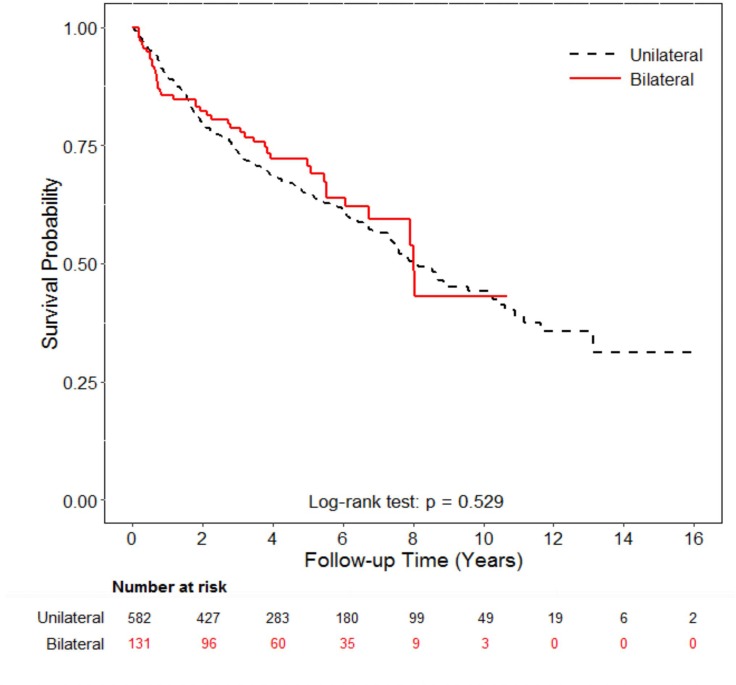
Overall survival analysis of patients with bilateral or unilateral nephron-ureterectomy The 5-year overall survival in bilateral NU group was 71% compared to 65% in unilateral NU group (*p* = 0.529).

We analyzed the overall survival between patients with or without NU and patients with different NU types. Patients who underwent NU had better 5-year overall survival (66% versus 51% in those without NU, *p* = 0.001, Figure 1). The 5-year overall survival in bilateral NU group was 71% compared to 65% in unilateral NU group (*p* = 0.529, Figure 2). To confirm the result, we also used Cox proportional model to compare the overall survival with adjustement for sex, age at cancer diagnosis, income, urbanization, and co-morbidities. Patients with unilateral and bilateral NU had significant adjusted HRs of 0.73 (95% CI: 0.59–0.91) and 0.67 (95% CI: 0.47–0.96) compared to non-NU group, respectively (Table 3). However, when we compared the OS by different NU types, the adjusted HR in OS of those with bilateral NU was 0.95 (95% CI: 0.68–1.32) compared to those with unilateral NU. Patients in different age groups (less or older than 65 years old), there were both similar 5-year OS between unilateral and bilateral NU groups (younger age: 72% versus 69% in unilateral NU, *p* = 0.5, older age: 52%% versus 77% in unilateral NU, *p* = 0.164). Similarly, patients with open and laparoscopic methods both had significant lower risk with HRs of 0.74 (95% CI: 0.59–0.93) and 0.67 (95% CI: 0.50–0.90) compared to those without NU with adjustment for confounding factors, respectively (Table 3). When comparing different surgical methods, the adjusted HR of OS in the laparoscopy method was 0.95 (95% CI: 0.73–1.25) compared to the open method (data not shown). In short, the OS was not different by NU types or by surgical methods. We also analyzed the post-NU chemotherapy and radiation therapy. The percentage of chemotherapy after NU in patients with unilateral and bilateral NU were 27.8% and 36.6%, respectively. About the radiation therapy, 11.9% of unilateral NU group and 13.7% in bilateral NU group had post-NU radiation therapy.

**Table 3:** Comparison of overall survival and bladder recurrence by different nephroureterectomy and surgical methods

	No. of Patients	Event	No. of Person-Years	IR (95% CI)	Hazard Ratio			
					Crude HR	95% CI	Adjusted HR*	95% CI
**Mortality**								
Nephroureterectomy								
Non-NU	218	139	1193.14	115.7 (97.9–136.7)	1.00		1.00	
Unilateral	582	241	2722.77	88.5 (78.0–100.4)	0.73	0.59–0.90	0.73	0.59–0.91
Bilateral	131	43	536.42	80.2 (59.5–108.1)	0.64	0.45–0.91	0.67	0.47–0.96
Methods								
Non-NU	218	139	1193.14	115.7 (97.9–136.7)	1.00		1.00	
Open	491	208	2445.96	85.0 (74.2–97.4)	0.71	0.57–0.88	0.74	0.59–0.93
Laparoscopic	222	76	813.23	93.5 (74.6–117)	0.73	0.55–0.97	0.67	0.50–0.90

IR: Incidence rate (per 1000 person-years), HR: Hazard ratio; NU, nephroureterectomy.

*HRs were adjusted for sex, age at cancer diagnosis, income, urbanization, and co-morbidities including diabetes, hypertension, tuberculosis, hyperlipidemia, chronic hepatitis B, chronic hepatitis C, and glomerulonephritis.

## DISCUSSION

UTUC is rare, representing only 5% of all urothelial tumors in western country [15]. However, the proportion markedly increased to 24–48% in ESRD patients with following kidney cancer [3]. In Taiwan, its incidence is much higher, especially among those on dialysis [11, 16]. NU represents the primary management for patient in dialysis with UTUC. There are some studies on the outcomes of NU for ESRD patients with UTUC [13, 14, 17], but the sample sizes are limited. A recent population-based study has shown that patients with ESRD and kidney cancer mainly renal cell carcinoma are at higher risk of in-hospital mortality and complications after nephrectomy [18]. But evidence supporting the safety and efficacy of NU for ESRD with UTUC remains unclear especially those of bilateral NU. In this study, we had confirmed the patients with ESRD and following UTUC in NU group had better 5-year overall survival and significant HR after adjustment for confounding factors when compared to non-NU group. The impact of NU was consistent no matter NU types or surgical methods.

Because there is a high recurrence rate of UTUC in patient on dialysis, Wu et al. have suggested that total urinary tract exenteration is a therapeutic option [13, 17]. In contrast, Kang et al. report that no significant difference is observed in terms of peri-operative mortality and OS between patients receiving one-stage or two-stage bilateral NU [14]. However, these results were based on experience of single institute. To date, this is the first national cohort study on clinical outcomes of NU for UTUC in patients on dialysis. In our study, there are 713 ESRD patients in NU group. The findings here reveal no significant difference in CCI between the bilateral and unilateral NU groups, which means similar peri-operative risks in both groups. Considering one-stage bilateral NU, there are no significant differences in 30- and 90-day mortality and duration of hospitalization compared to unilateral NU. These results confirm that bilateral NU is safe and there is no additional surgical risk compared to unilateral NU.

Furthermore, among the 582 patients who underwent unilateral NU, 115 (19.7%) subsequently underwent contralateral UN with a mean interval period of 695 days. That means there was nearly one-fifth chance of each ESRD patient with UTUC has to take high surgical risk for the successive NU within two years.

Whether laparoscopic NU offers reliable peri-operative safety to patients on dialysis lacks solid evidence. Only few case series have been published about laparoscopic NU for ESRD patients with renal tumors [19]. This population-based study further confirms that laparoscopic NU is feasible for patients on dialysis based on similar 30- and 90-day mortality and 2.7 days less hospitalization compared to the open method.

Owing to the advances of hemodialysis with growing elderly population, there is a critical need for treatment options for elderly ESRD patients (≥ 65 years old). Some vascular and colon diverticular studies suggest to avoid elective surgical intervention for this population due to the high mortality and morbidity [20, 21]. However, the poor outcome of NU for elderly with ESRD and UTUC is unclear. In considering elective surgery for elderly ESRD patients, some surgeons suggest non-operative management, not only because surgical intervention may not benefit patients with limited life expectancy but also because of significantly elevated risk of post-operative complications [20]. This may explain that the higher proportion of elderly in non-NU group in our study. Such beliefs arose from a 1993 study, which revealed that patients with ESRD who are 65–79 years of age have a five-year survival rate of only 19% [21]. However, with advances in hemodialysis, the 5-year survival rate has much increased [22]. Thus, these concepts need to be revisited in terms of applicability to elderly ESRD patients. The 30- and 90-day mortality in elderly is 7/257 (2.7%) and 17/257 (6.6%), respectively. On the other hand, there is no increase in 30- and 90-day mortality in bilateral NU compared to unilateral NU among the elderly. In addition, compared to younger patients, elderly ESRD patients who have undergone NU have similar duration of hospitalization. Thus, we suggest that whenever possible, NU remains the primary treatment option for elderly ESRD patients with UTUC. As regards one-stage bilateral NU for elderly ESRD patients, the limited case number (*n* = 32) still showed similar safety. In patients younger than 65 years old, the 30-day mortality were zero in both unilateral and bilateral NU groups. But those with bilateral NU showed worse 90-day mortality. However, because the number of mortality was limited, the small number difference may lead to statistical significance. In addition, this data was all-cause mortality rather than cancer specific mortality. Most importantly, the 5-year overall survival between unilateral and bilateral NU groups was similar (72% versus 69%, *p* = 0.5, respectively).

Active surveillance has been suggested because of higher risk of in-hospital mortality in ESRD patients undergoing nephrectomy for non-metastatic RCC [18]. However, there are basic difference in the anatomic characteristics between RCC and UTUC. Patient may have a larger tumor size in RCC, thereby requiring more extensive surgery. On the other hand, ESRD patients with the atrophic kidneys require less extensive surgery for UTUC. Patients who underwent NU has better five-year overall survival rate (66% versus 51% in those without NU, *p* = 0.001). Thus, aggressive nephrectomy for ESRD patients with UTUC is still recommended as the primary treatment.

This study has certain limitations. First, the diagnoses of ESRD, UTUC, and other co-morbidities are based on ICD-9 (International Classification of Diseases, Ninth Revision) codes, so misclassification is possible. Nonetheless, the use of ICD-9 codes for patients with chronic diseases has been validated in previous national cohort studies and the NHI Bureau of Taiwan reviews the charts and imposes heavy penalties for unsuitable charges or malpractice to ensure accurate coding [23]. To further reduce this issue, the ESRD and UTUC diagnosis were taken from the Catastrophic Illness Patient Database [24]. Second, the database does not include important tumor characteristics, such as stage and grade. However, according to previous studies [13, 14], most patients with ESRD and UTUC had early stage and there were no metastasis at initial diagnosis in both total 70 and 73 study subjects. There were even rare patients with stage T4, only 3 (4.3%) in 70 and the other 1 (1.4%) in 73 patients. However, most of these patients had high tumor grading. There were 82.9% and 94.5% of patients with grade 2 or 3 in these two studies, respectively. We supposed that most patients with ESRD and following UTUC always have characteristics of early stage but high-grade tumor. In this study, we mainly compared the safety and efficacy of different surgical methods for this population. Hence, we assumed that enrolled patient all had resectable tumor. Third, the number of patients who underwent laparoscopic NU is invariably lower than that for open NU. Therefore, the good result of laparoscopic NU can be attributed mainly to a bias in the data from thelaparoscopic NU study arms [25].

## MATERIALS AND METHODS

### Data source

Since March 1995, the Taiwanese government has been implementing the National Health Insurance (NHI) program, which provides general health insurance coverage to almost the entire Taiwanese population. The National Health Insurance Research Database (NHIRD) of this program contains registration files and original claims data for reimbursement and is maintained by the National Health Research Institute (NHRI) and is provided to scientists for research purposes since 2000. The NHIRD has detailed healthcare data of 25.68 million enrollees (99% of the population) based on a random sample of all enrollees of the NHI program. In this population-based retrospective study, study subjects were obtained from the Registry for Catastrophic Illness Patient Database (RCIPD), a subsection of the NHIRD.

### Study subjects

All Taiwanese patients with ESRD and on dialysis are included in the RCIPD. Additional requirement for inclusion are cytologic or pathology reports or evidence from additional laboratory and imaging studies supporting the diagnosis of cancer. Using the diagnostic variables in RCIPD covering the period 1997–2013, ESRD patients with UTUC were selected from the NHIRD using the ICD-9-CM codes 189.1, 189.2 (for UTUC) and 585 (for ESRD) for identification. All subjects who were newly diagnosed with UTUC after ESRD diagnosis between January, 1998 and December, 2012 were included. All medical records were extracted from the NHIRD and analyzed. The enrolled subjects were followed-up until death or the end of 2013.

Those with erroneous or missing data were excluded, as well as those previously diagnosed of any cancer before ESRD and UTUC diagnosis. Those with synchronous lower urinary tract urothelial carcinoma other than UTUC were also excluded. Total 931 subjects were included in the final analysis. We classified these patients by either NU or not, NU types and surgical methods. For NU types, patients with unilateral surgical resection of UTUC were classified as unilateral NU group. Patients with bilateral UTUC and receiving bilateral NU in the same time were defined as one-stage bilateral NU group. On the other hand, patients with surgical NU and no matter unilateral or bilateral UTUC were classified into either open method or laparoscopic method groups. This study was approved by the institutional review board at the Chang Gung Memorial Hospital.

### Statistical analysis

Patients with death record in the RCPID or admission database of NHIRD were defined as death event. Cox proportional hazards models were used to compute the hazard ratios (HRs) and 95% confidence intervals (CIs) of death, after adjustments for type of NU (unilateral or bilateral), surgical methods (open or laparoscopic), age, sex, Charlson co-morbidity index (CCI), and socio-demographic characteristics (i.e. income and level of urbanization).

The Kaplan-Meier method was used to calculate the survival curves, while the log-rank test was used to compare differences in survival curves for patients with bilateral and unilateral NU. All statistical analyses were conducted using the SAS statistical software (version 9.4; SAS Institute, Cary, NC, USA).

## CONCLUSIONS

This population-based study of ESRD patients with UTUC revealed that NU provides significant five-year survival benefits. One-stage bilateral NU both provides comparable safety profile and avoids 19.7% of successive contralateral NU in less than two years. Even for the elderly, one-stage bilateral NU is safe and feasible.
